# Long non-coding RNA MALAT1 protects preterm infants with bronchopulmonary dysplasia by inhibiting cell apoptosis

**DOI:** 10.1186/s12890-017-0524-1

**Published:** 2017-12-13

**Authors:** Cheng Cai, Jiajun Qiu, Gang Qiu, Yihuan Chen, Zhijun Song, Juan Li, Xiaohui Gong

**Affiliations:** Department of Neonatology, Shanghai Children’s Hospital, Shanghai Jiao Tong University, Shanghai, People’s Republic of China 200062

**Keywords:** BPD, Preterm neonates, Hyperoxia, MALAT1, Apoptosis

## Abstract

**Background:**

Bronchopulmonary dysplasia (BPD) is a neonatal chronic lung disease characterized by impaired pulmonary alveolar development in preterm infants. Until now, little is known about the molecular and cellular basis of BPD. There is increasing evidence that lncRNAs regulate cell proliferation and apoptosis during lung organogenesis. The potential role of lncRNAs in the pathogenesis of BPD is unclear. This study aims to clarify the role of MALAT1 during the process of BPD in preterm infants and illustrate the protective effect of MALAT1 involved in preterm infants.

**Methods:**

We assessed the expression of MALAT1 in BPD mice lung tissues by reanalyzing dataset GSE25286 (Mouse GEO Genome 4302 Array) from gene expression database gene expression omnibus (GEO), and verified MALAT1 expression in BPD patients by realtime q-PCR. Then the role of MALAT1 in regulating cell biology was examined by profiling dataset GSE43830. The expression of CDC6, a known antiapoptopic gene was verified in BPD patients and the alveolar epithelial cell line A549 cells in which MALAT1 was knocked down. Cell apoptosis was determined by FACS using PI/Annexin-V staining.

**Results:**

The expression of MALAT1 was significantly evaluated in lung tissues of BPD mice at day 14 and day 29 compared to WT (*P* < 0.05). In consistent with mRNA array profiling analysis, MALAT1 expression level in blood samples from preterm infants with BPD was significantly increased. Bioinformative data analysis of MALAT1 knockdown in WI-38 cells showed various differentially expressed genes were found enriched in apoptosis related pathway. Down-regulation of antiapoptopic gene, CDC6 expression was further verified by q-PCR result. PI/Annexin-V apoptisis assay results showed that MALAT1 knocked down in the alveolar epithelial cell line (A549) promotes cell apoptosis.

**Conclusions:**

In our study, we found that up-regulation of lncRNA MALAT1 could protect preterm infants with BPD by inhibiting cell apoptosis. These data provide novel insights into MALAT1 regulation which may be relevant to cell fate and shed light on BPD prevention and treatment.

**Electronic supplementary material:**

The online version of this article (10.1186/s12890-017-0524-1) contains supplementary material, which is available to authorized users.

## Background

The bronchopulmonary dysplasia (BPD), neonatal form of chronic lung disease, remains major threat to premature infants nowadays. Accompanied with the rapid development of perinatal medicine which makes the incidence of premature infants is increasing. Extreme immaturity results in incomplete development of organs and respiratory insufficiency remains the major contributor to perinatal morbidity and mortality [1].Supplemental oxygen can be life-saving in preterm infants, but may also increase the risk of getting BPD. BPD can be developed into multi-organ disorder, which affects up to 50% of extremely low birth weight infants (ELBWI) <1000 g [2]. Most of extremely preterm infants are in the saccular stage of lung development. BPD is caused by the dysfunctional of lung development in this critical period. The diagnosis is associated with lifelong long-term pulmonary problems and abnormal neurodevelopment outcome [3]. However the underlying pathogenesis is not fully understood and few evidence-based strategies to prevent or treat BPD are currently available.

Oxidative stress is considered as contributor of oxygen radical disease in neonates including necrotizing enterocolitis (NEC), intraventricular hemorrhage (IVH), periventricular leukomalacia (PVL), retinopathy of prematurity (ROP) and neonatal BPD [4] . Because premature infants have little antioxidant protection, they are particularly prone to oxygen free radical damage [5]. In particular, these premature infants often need to receive oxygen therapy after birth. Oxidative stress activates inflammatory cells and increases proinflammatory cytokines, and causing damage to the respiratory tract epithelium and inactivating surfactant [6]. Above all, oxidative stress induced cell death plays a very important role in the occurrence and development of BPD in premature infants.

Long non-coding RNAs (lncRNAs) are a newly defined class of non-coding RNAs with length greater than 200 nucleotides, and play important roles in various biological processes including cell proliferation, cell death, oxidative stress resistance [7]. Previous studies have found that MALAT1 is significantly highly expressed in non–small cell lung carcinoma (NSCLC) patients and regulates invasion, migration, and tumor growth in many other cancer types [8]. However, the mechanism by which functional MALAT1 modulates the pathogenesis of BPD is not well understood. We report here that MALAT1 is up-regulated in BPD newborn mice and BPD patients, and MALAT1 knockdown induce apoptosis in A549 cell. Our results may reflect an important role for MALAT1 during the development of BPD. Therefore, this study of MALAT1 has important clinical value for the protective effect of BPD in premature infants.

## Methods

### GEO bioinformatics

Gene chip datasets which this study used in the bioinformatics analysis were both downloaded from GEO (Gene Expression Omnibus). Dataset GSE25286 was based on lung tissue from wild type (WT) (*n* = 4) and BPD Mice (*n* = 6) with two time points (Day 14 and Day 29) [9].The mice model of hyperoxia-induced bronchopulmonary dysplasia was described in previous paper [9]. Dataset GSE43830 used WI38 cell (human diploid lung fibroblasts) in which MALAT1 was knocked down as materials, comparing their gene expressions with normal WI38 cell (knocked down samples: 4, control samples: 2) [10]. The differential expression analysis was done by Transcriptome Analysis Console software. The differential expressed genes were defined as (Fold Change < −2 or Fold Change >2, ANOVA *p*-value <0.05). We used the minus sign to indicate the down-regulation. The KEGG enrichment analysis was done by R package clusterProfiler [11].

### Subjects and sample collection

A total of 20 premature infants with BPD according to the National Institute of Child Health and Human Development (NICHD) guidelines [12] and 20 non-BPD age-matched controls were enrolled from clinic at department of neonatology in Shanghai Children Hospital. The study was approved by the Ethics Committee of the Shanghai Children Hospital. Human peripheral blood samples were obtained from these patients and written informed consent was obtained from the guardians of the patients.

### Total RNA isolation and Realtime q-PCR verification

Total RNA was isolated from patient blood samples (BPD infants and controls) using TRIzol reagent (Invitrogen, Carlsbad, USA) according to the manufacturer’s instructions. RNA was quantified using NanoDrop ND-2000 Spectrophotometer (NanoDrop Wilmington DE). Reverse transcription (RT) reactions and real-time PCR were carried out as we previously described [13]. The relative expression of MALAT1 and CDC6 compared to β-Actin was calculated with the 2-ΔΔCt method. Primers are listed in Table 1.

**Table 1:** Primers of MALAT1, CDC6 and β-Actin

Gene name	Primer	Sequence(5′-3′)
MALAT1	Forward	CTATGCTGTTGGCACGACA
	Reverse	TCCTGAGGTGACTGTGAACC
β-Actin	Forward	TCTGTGTGGATTGGTGGCTCTA
	Reverse	CTGCTTGCTGATCCACATCTGCTCCTCGTGTAA
CDC6	Forward	AAGCCCTG
	Reverse	TCAAATACCAATCTTCGTCCC
β-Actin(H)	Forward	’GTGGCCGAGGACTTTGATTG
	Reverse	CCTGTAACAACGCATCTCATATT

### Cell culture

A549 cells (Human Type II alveolar epithelial cells) (ATCC, CCL-185, USA) were grown in Roswell Park Memorial Institute medium (RPMI 1640) (Gibco; Carlsbad, CA, USA) supplemented with 10% fetal bovine serum (FBS) (Gibco) in 5% CO_2_ at 37 °C.

### Measurement of cell apoptosis by flow cytometry

A549 cells transfected with shRNA of MALAT1 were plated in six-well plates. After 24-h incubation, apoptosis was measured by ApopNexin™ fluorescein isothiocyanate (FITC) Apoptosis Detection Kit (APT750, Millipore, Temecula, CA) as previously described. Fluorescence due to FITC and PI staining was measured in a flow cytometer (Cytomics FC 500, Beckman Coulter, Brea, CA).

### Statistics

Experimental data were analyzed with the Student’s t-test. t-test: *, *p* < 0.05. * *, *p* < 0.01.Throughout the paper, values are represented as the mean ± standard deviation of at least 3 independent experiments.

## Results

### LncRNA MALAT1 is significantly up-regulated in the lung tissue of BPD mice

**Fig. 1:**
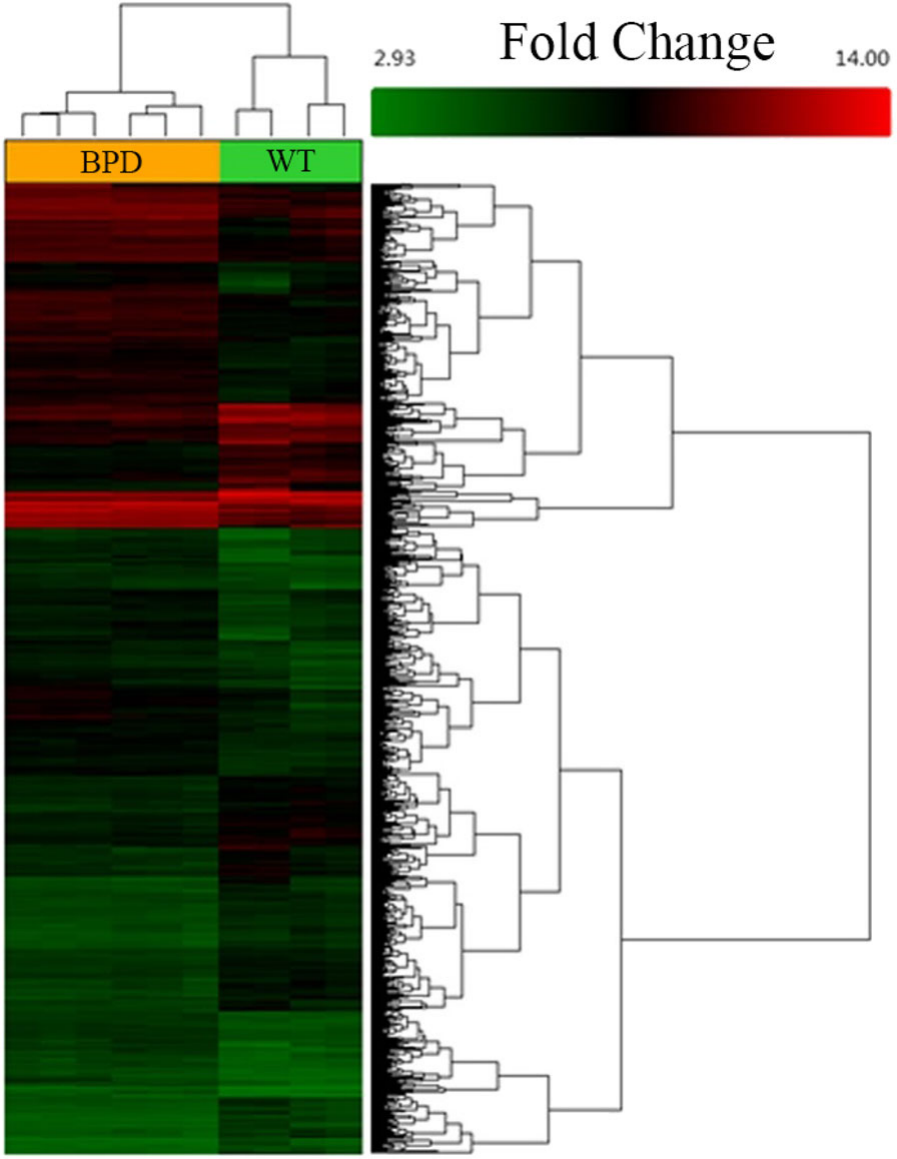
Heat map of differentially expressing genes in GSE25286 The GEO dataset GSE25286 which includes 10 BPD and WT mice lung samples at two time points [9], was subjected to bioinformatic analysis for differential gene expression pattern. The heat map shows results from analysis with cut-off *p* value < 0.01 and fold change ≥ 2. The *x* axis represents the samples, and genes are shown on the *y* axis. Red spots represent high-expressing genes, and green spots represent low-expressing genes. The sample types are shown with bar colors in the dendrogram; yellow stripes represent lung tissue from BPD mice and, green stripes are lung tissue from WT mice.

**Fig. 2:**
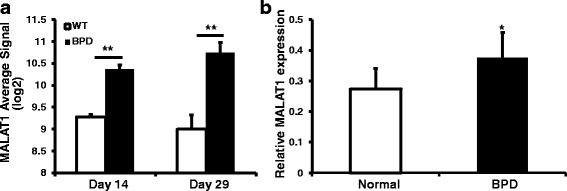
MALAT1 expression level in BPD murine model and patients. **a** MALAT1 expression differed between BPD and WT mice at Day 14 and Day 29. Y-axis showed normalized signal of MALAT1 in dataset GSE25286. The differential expresseion (*) was defined as (Fold Change < −2 or Fold Change >2, ANOVA *p*-value <0.05). **b** MALAT1 expression differed between BPD and normal patient. Y-axis showed normalized relative expression level of MALAT1. *, *P* < 0.05

According to GSE25286 dataset, microarray profiling revealed 1616 differentially expressed genes, with 962 up-regulated and 654 down-regulated genes in the BPD mice lungs compared to the WT mice (Fig. 1 and Additional file 1: Table S1). Surprisingly, MALAT1 was the only lncRNA found abundant in BPD mice than in WT mice based on GSE25286 (Table 2). Next, we compared MALAT1 expression between BPD and WT mice at two time points (Day 14and Day 29 mice) respectively. The results showed that MATAL1 expression was significantly up-regulated in BPD mice at both Day 14 Mice (*p* < 0.001) and Day 29 Mice (*p* = 0.005) (Fig. 2A).

**Table 2:** Probe Cluster of MALAT1 which differential expressed between BPD Mice and WT

Probe Cluster ID	Gene Symbol	BPD Avg Signal(log2)	WT Avg Signal(log2)	Fold Change	ANOVA p-value	FDR p-value
1436202_at 1452378_at	MALAT1 MALAT1	10.48 9.12	9.26 8.02	2.33 2.14	0.000028 0.006168	0.000788 0.022612

### Realtime q-PCR verification of MALAT1 expression in BPD patients.

We thus verified the GEO dataset result by q-PCR verification of MALAT1 in peripheral blood samples from BPD patients. To our notice, the expression of MALAT1 was also significantly increased in premature infants with BPD, compared with normal premature infants (Fig. 2B).

### Knockdown MALAT1 in WI-38 cells promotes cell apoptosis

**Fig. 3:**
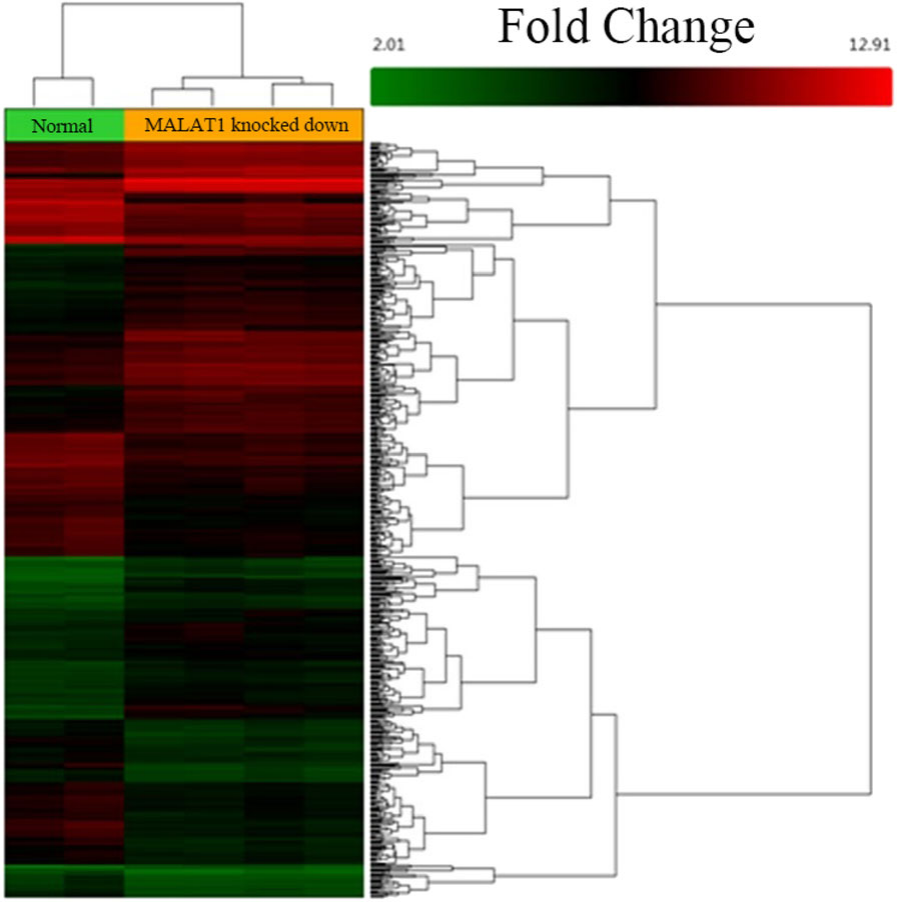
Heat map of differentially expressing genes in GSE43830. Heat map shows results from analysis with cut-off p value < 0.01 and fold change ≥ 2. The *x* axis represents the samples, and genes are shown on the *y* axis. Red spots represent high-expressing genes, and green spots represent low-expressing genes. The sample types are shown with bar colors in the dendrogram; yellow stripes represent WI-38 cells in which MALAT1 was knocked down, green stripes are normal WI-38 cells

**Fig. 4:**
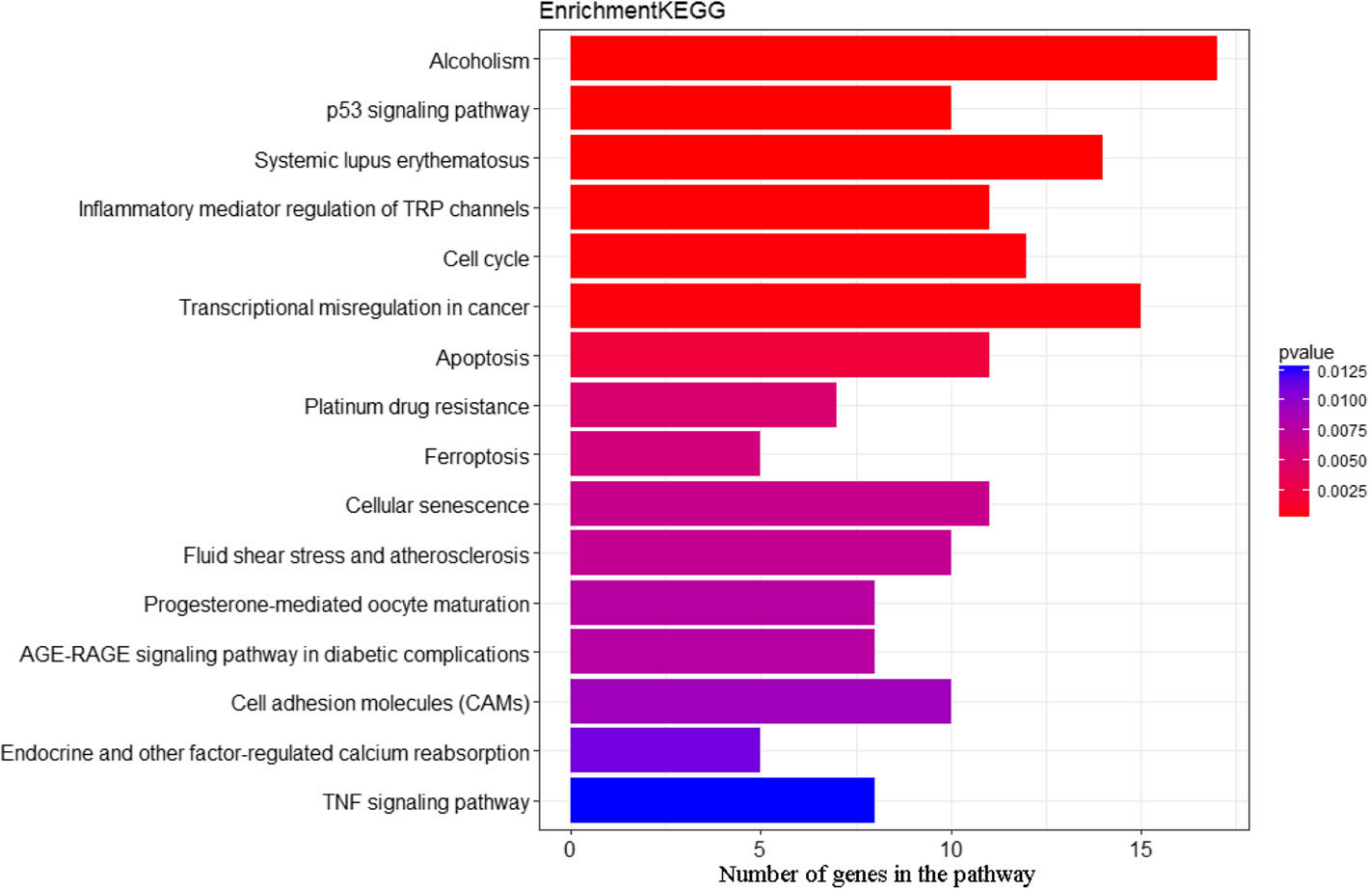
KEGG enrichment results of differentially expressed genes in GSE43830. Graphic output represents differentially expressed genes by KEGG enrichment analysis. The blue to purple color bars represent enrichment level of genes in different pathway with statistical differences

**Fig. 5:**
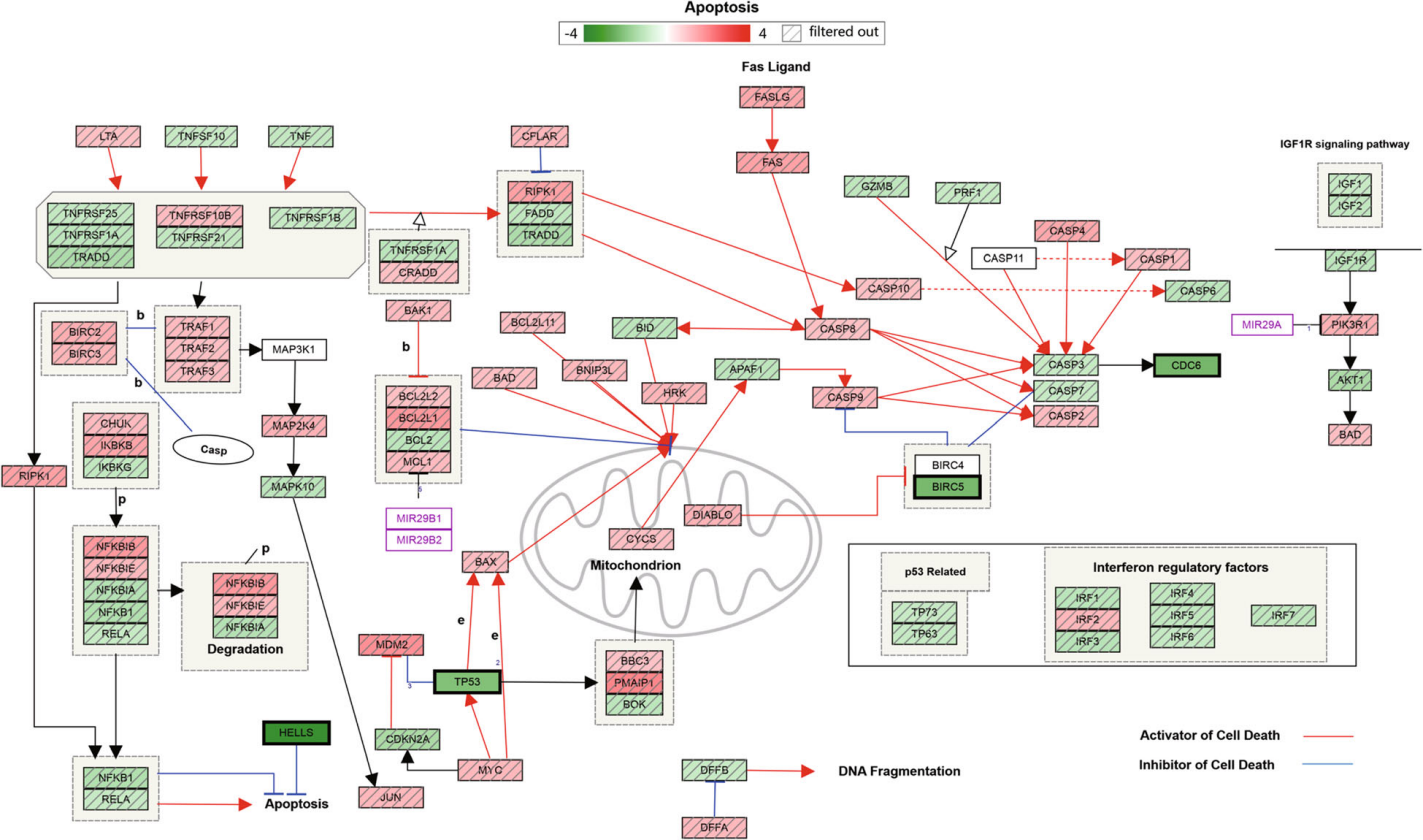
Differential expressed genes distribution in “Apoptosis” The fold change value are shown with bar colors in the dendrogram; red color represents up-regulate, green color means down-regulate. The red arrows indicated the genes were activator of cell death, while the blue arrows indicated the genes were inhibitor of cell death

According to GSE43830 dataset result, RNA-seq revealed 570 differentially expressed genes, with 305 up-regulated and 265 down-regulated genes in lncRNA MALAT1depleted WI-38 (human diploid lung fibroblasts) cells compared with WT (Fig. 3 and Additional file 2: Table S2). To identify the biological pathways that are active in the MALAT1 depleted WI-38 cells, we mapped the genes to the heat map. To our notice, the result of KEGG pathway enrichment analysis confirmed that the differentially expressed genes do enrich in apoptosis pathway (*P* = 0.002) (Fig. 4). Furthermore, we found a number of differentially expressed genes are distributed in apoptosis related pathway (Fig. 5). MALAT1 knockdown in WI-38 cells significantly decreased expression of CDC6 (FC = −2.37, *P* = 0.040) and q-PCR results in peripheral blood samples of BPD patient verified above data (*P* = 0.017).(Figure4). Taken together, MALAT1 down-regulation showed induction of cell apoptosis in WI-38 cells.

**Fig. 6:**
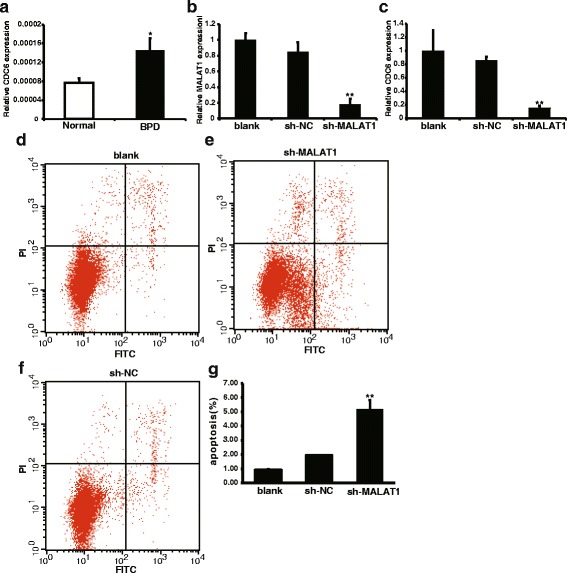
MALAT1 silencing induced apoptosis in A549 cells. **a** q-PCR verification of CDC6 expression in BPD patients compared with normal. Y-axis showed normalized relative expression level of CDC6. *, *P* < 0.05. **b** A549 cells were transfected with shMALAT1 or shRNA negative control, and CDC6 expression level was compared among blank (no shRNA), sh-NC (shRNA negative control_and sh-MALAT1 (MALAT1 shRNA).Y-axis showed normalized relative expression level of CDC6. **, *P* < 0.01. PI/Annexin-FITC apoptosis assay. The apoptosis rate of Blank, sh-NC and sh-MALAT1 was (0.94 ± 0.03), (1.96 ± 0.01) and (5.18 ± 0.65), respectively. Profiles of (**c**-**e**) are representative of at least three independent experiments. Statistical analysis is shown in (**f**) (*p* < 0.01)

### Silencing of MALAT1 promotes cell apoptosis in A549cells

We next investigated whether MALAT1 knockdown in A549 cells could also affect apoptosis. The apoptosis levels of A549 cells transfected with shMALAT1 or shRNA negative control were analyzed. FACs results showed that inhibition of MALAT1 expression can induce apoptosis (Fig. 6). Thus, these results indicate that down-regulation of MALAT1 can promote apoptosis levels in A549 cells.

## Discussion

To date, little is known on the function of lncRNA MALAT1 in murine model and patients with BPD and its possible role during BPD development process. We found significantly higher expression of MALAT1 in lung tissue of BPD mice model compared to WT mice. Further, we demonstrate that MALAT1 is differentially regulated in peripheral blood cells from patients with BPD compared to normal ones. In addition, we identified deregulation of apoptosis related genes in WI-38 cells with MALAT1 depletion. Our data also showed a significant increase in apoptosis in A549 cells when MALAT1 was knocked down.

In last two decades, with the rapid development of perinatal medicine, the survival rate of premature infants of very low birth weight infants (VLBWI) and extremely low birth weight infants (ELBWI) has significantly improved [14]. Of all births in the US, babies born at <32 weeks gestation account for 1.9% of live births, resulting in more than 75,000 babies admitted to neonatal intensive care unit (NICU) each year [15]. Despite many advances in neonatal ventilation techniques, widespread use of surfactant and antenatal corticosteroids, the incidence of bronchopulmonary dysplasia (BPD) has remained the same or even increased slightly.

The development of premature lung is immature, 30%–50% premature infants with gestational age less than 32 weeks premature need to use a variety of oxygen therapy after birth, such as oxygen inhalation, the humidified high flow nasal cannula (HHFNC) and nasal continuous positive airway pressure (NCPAP), or mechanical ventilation treatment which provide high concentration oxygen exposure opportunities and very easily lead to lung injury [16]. BPD has become one of the most difficult problems in NICU. BPD is the most common chronic respiratory disease in infants and a devastating condition that disrupts the developmental process of the lung secondary to preterm birth [17].In the US, BPD is the leading cause of chronic lung disease (CLD) in babies and the third leading cause in children, but the exact mechanism of BPD has not been fully elucidated.

Long non-coding RNA (lncRNA) refers to a class of non-coding RNA longer than 200 nucleotides and devoid of an open reading frame that can be translated into a protein. LncRNA was recently demonstrated to play functional roles in the regulation of gene expression by means of dosage compensation, imprinting, transcriptional regulation and nuclear organization. MALAT1 named after its initially discovered function, is lncRNA of over 8000 bp [18]. MALAT1 was later identified to be anunclear enriched abundant transcript [19] and expressed in the lungs, pancreas, nerve system and other healthy organs [18, 20]. Abnormal expression of MALAT1 has also been detected in various types of cancers, including lung cancer, endometrial stromal sarcoma, hepatocellular carcinoma, breast cancer and pancreatic cancer [21, 22].

Over past years of research, there has been reorganization of lncRNA involvement in gene silencing and playing an important role in a variety of biological processes [23]. Our study found that lncRNA MALAT1expression was up-regulated in lung tissue of BPD mice at Day 14 and Day 29 compared with WT mice according to microarray data set GSE25286. The q-PCR verification results showed the expression of MALAT1 in peripheral blood of preterm infants of BPD was also up-regulated compared with normal premature infants. Taken together, the experimental results show that MALAT1 should play an important role in protecting the occurrence and development process of BPD.

To clarifying MALAT1 function in regulating detailed cell biological process, we analyzed differentially expressed gene distribution in WI-38 cells with MALAT1 depletion. Cell division cycle 6 (CDC6) is required for the initiation of DNA replication protein, its main function is to participate in the “pre replication complex assembled (pre-RC)” [24]. The inhibition mechanism of CDC6 induced apoptosis has not been fully elucidated. Under normal circumstances, CDC6 enter the nucleus pre-RC the assembly will be cyclin dependent kinase 2 (CDK2) phosphorylation and transported out of the nucleus. Recent studies have found that CDC6 itself has a role in inhibiting apoptosis, complex CDC6 through its ATPase domain and apoptosis protease activating factor Apaf-1 to form stable, blocking the formation of apoptotic bodies [25]. Previous study indicated that cell apoptosis plays an important role in the occurrence and development process of premature BPD [26]. Our study showed that MALAT1 depletion in WI-38 cells induces cell apoptosis. The expression level of CDC6 was down-regulated. Furthermore, q-PCR results of BPD patients and MALAT1 silencing in A549 cells verified WI-38 data. Moreover, the results of A549 cells apoptosis analyzed by flow cytometry showed that, after MALAT1 was knocked down, apoptosis in A549 cells was significantly increased. As mentioned above, our data suggests MALAT1 participate in an important role in preterm BPD infants.

There are some limitations to our study. Ideally, we would like to confirm RNA-Seq data obtained in WI-38 cells in primary human alveolar type II cells instead of A549 cells. However, such cells are difficult to obtain and cultured for their requirement of fresh isolation from human lung tissue. Although rodent type II cells are more readily available, the functional differences from human type II cells are noteworthy. Furthermore, although we have demonstrated that the enhanced uptake of MALAT1 in these experiments is probably due to protect effect of inhibiting apoptosis; it is unclear whether this is the primary effect of MALAT1 on these cells. As such, the primary effect of MALAT1 on BPD remains to be elucidated.

## Conclusions

In conclusion, MALAT1 may protect preterm infants with BPD by inhibiting apoptosis, and provide a new strategy for the prevention and treatment of premature BPD.

## Additional files

Additional file 1: Table S1. List of differentially expressed genes in GSE25286 (XLSX 157 kb)

Additional file 2: Table S2. List of differentially expressed genes in GSE43830 (XLSX 56 kb)

## Abbreviations

BPD: Bronchopulmonary dysplasia
CDC6: Cell division cycle 6
CON: Blank Control Group
GEO: Gene Expression Omnibus
IVH: Intraventricular hemorrhage
KD: MALAT1 knock down Group
lncRNA: Long non-coding RNA
MALAT1: Metastasis associated in lung adenocarcinoma transcript 1
NC: Negative Control Group
NEC: Necrotizing enterocolitis
PVL: Periventricular leukomalacia
ROP: Retinopathy of prematurity
RT-PCR: Real time-PCR
